# Activation of Early Defense Signals in Seedlings of *Nicotiana benthamiana* Treated with Chitin Nanoparticles

**DOI:** 10.3390/plants9050607

**Published:** 2020-05-10

**Authors:** Miguel López, Elisa Miranda, Cecilia Ramos, Héctor García, Andrónico Neira-Carrillo

**Affiliations:** 1 Department of Biological Animal Sciences, Faculty of Veterinary and Animal Sciences, University of Chile, La Pintana, Santiago 8820808, Chile; mig.lopez@u.uchile.cl; 2 Department of Molecular Biology, Laboratorios Diagnofruit Ltda., Ñuñoa, Santiago 7770273, Chile; emiranda@diagnofruit.cl (E.M.); cramosb@udla.cl (C.R.); hgarcia@diagnofruit.cl (H.G.); 3 Núcleo de Investigaciones Aplicadas en Ciencias Veterinarias y Agronómicas (Nucleus of Applied Research in Veterinary and Agronomical Sciences), Universidad de las Americas, Campus Providencia, Santiago 7500972, Chile

**Keywords:** nanoparticles, elicitor, emulsion, early defense responses, PTI, chitin

## Abstract

Chitin is an excellent material for the synthesis of nanoparticles because it is an elicitor and can form nanostructured materials. The application of chitin nanoparticles (CNPs) in plants can activate early defense responses associated with chitin. In this study, CNPs were synthesized by water in oil (W/O) emulsion using an aqueous chitin solution. The CNPs were characterized and used to evaluate the activation of genes related to early responses to chitin and the production of reactive oxygen species (ROS) on seedlings of *Nicotiana benthamiana*. The CNPs had an average size of 280 nm in diameter, a polydispersity of 0.299, a surface charge of 26.9 mV, and their chemical composition was corroborated by the disappearance of microaggregated CNPs treated with chitinases observed under a microscope. Seedlings treated with CNPs for one hour revealed increments in the expression of genes *STZ*, *ATL2*, and *MAPK3,* in contrast when they were treated with chitin oligomers, and no changes in gene *CERK1* was detected in both conditions. Finally, the synthesis of ROS mediated by CNPs was detected in seedlings, which was higher than those generated by the treatment of chitin oligomers. These results demonstrated the capability to generate CNPs by emulsion, which are capable of triggering responses related to early defense in *N. benthamiana* more efficiently than chitin oligomers.

## 1. Introduction

The use of chitin, a natural polymer formed by units of N-acetylglucosamine, has proven to be suitable for treatment on crops and post-harvest management due to its ability to enhance productivity parameters in plants and fruits, as well as being biodegradable and biocompatible [1,2]. Chitin could be perceived by plants as a microbe-associated molecular pattern (often called pathogen-associated molecular pattern, MAMP or PAMP) because it is a structural component in the exoskeleton of arthropods and the cell walls of phytopathogenic fungi, classifying this molecule as an elicitor [3]. Others MAMPs are common structures, along with microorganisms such as lipopolysaccharide (LPS), peptidoglycans, dsRNA, or flagellin, that could activate molecular and physiological defense responses, called PAMP-triggered immunity (PTI), which acts as the first line of immunity when the host plant does not recognize other damage signals or specific pathogenic effectors [4].

The molecular mechanisms of plant defense mediated by chitin were previously elucidated, which begins with a recognition of chitin oligomers by the LysM family receptors, such as CERK1 and LYK5, in the plasma membrane of plant cells [5,6], followed by the activation of an intracellular signaling cascade controlled by mitogen-activated protein kinases (MAPK), which generates responses like the synthesis of reactive oxygen species (ROS) or activation/repression of transcription factors and expression of genes related to defensive responses against pathogens [7,8,9]. This mechanism has been described in *Arabidopsis thaliana* [3,7,10,11,12], *Lotus japonicus* [13], *Medicago truncatula* [14], and *Oryza sativa* [9,15,16].

Some of the physical-chemical characteristics of chitin mean this material is difficult to handle, which has promoted the study of its chemical derivatives as chitosan or hydrolyzed chitin. However, as chitin is a polymer, it may be possible to use it for the development of advanced nanotechnological applications [17]. The basis of nanotechnology is the development of nanostructured materials, or nanomaterials, defined as materials structured on nanometric scales, providing them some special characteristics that are not present on its micro and macro scales. Those characteristics are dependent on their size, chemical composition, or surface modifications [18]. In agriculture, the use of nanotechnology has allowed for the optimization of processes necessary for an accurate agricultural management plan, such as the delivery of molecules with biological activity, nano-clays for soil remediation, or nanosensors for the detection of chemicals and microorganisms, among several others [19]. Nanoparticles are one of the most developed nanomaterials, the characteristics of which are determined by their composition and chemical nature. In this context, chitin is an excellent material for the synthesis of nanoparticles due to its ability to generate nanomaterials and to be an elicitor, allowing chitin nanoparticles (CNPs) to activate defense mechanisms in plants.

In this study, CNPs were synthesized using a chitin solution under an emulsion water in oil (W/O) to form a nanostructured material capable of activating early defensive responses in seedlings of *Nicotiana benthamiana*. Our results indicated that the CNPs obtained by emulsion are capable of initiating signals related to the recognition of chitin in plants, such as the expression of early genes regulated by chitin and the synthesis of ROS more efficiently than chitin oligomers. These results suggest that CNPs obtained by emulsion possess an elicitory activity in plants, more improved than chitin oligomers, and that enhances the versatility and possibly the use of this chitin-based nanomaterial for future agricultural applications.

## 2. Results

### 2.1. CNPs Can Be Synthesized by Emulsion W/O

**Figure 1:**
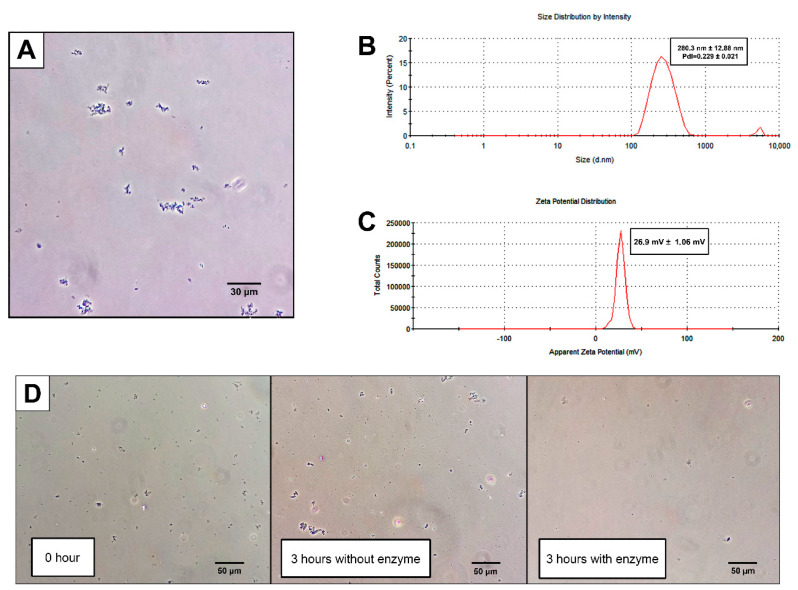
Characterization of the chitin nanoparticles (CNPs) synthesized by emulsion. (**A**) A micrograph of a suspension of CNPs under a phase-contrast optical microscope. The CNPs could microaggregate in the aqueous solution. (**B**) The determination of the size of the CNPs, determined by dynamic light scattering. The size dispersion of CNPs is between approximately 100 to 800 nm in diameter, with an average peak at 280.3 nm. (**C**) The surface charge of the CNPs, determined by electrophoretic light scattering. This charge is 26.9 mV, indicating a positive surface. (**D**) The enzymatic digestion of CNPs with chitinases. At the initial time, microaggregated CNPs can be observed (left square) and were held in suspension after 3 h of incubation without enzyme (center square). After 3 h of incubation with chitinase, the microaggregated CNPs had a drastic decrease in their concentration (right square).

The chitin solution (CS) was obtained by frozen-thawing and had a concentration of 9.15 mg/mL (0.915% chitin starting from a 2% suspension). This solution was used to obtain CNPs by emulsion W/O, resulting in a cloudy suspension formed by microaggregates visible under phase-contrast microscopy (Figure 1A). The final size of the CNPs was 280.3 ± 12.88 nm in diameter, with a polydispersity of 0.229 ± 0.021 and a Z-potential of 26.9 ± 1.6 mV (Figure 1B,C). After the incubation of the CNPs with chitinases, a decrease in the microaggregates formed by the CNPs was observed as opposed to the CNPs incubated without the enzyme (Figure 1D), which indicated that the chitin-degrading enzymes recognize these microaggregates as chitin structures.

### 2.2. CNPs Induce Expression of Genes Activated by Chitin Recognition in Nicotiana benthamiana

**Figure 2:**
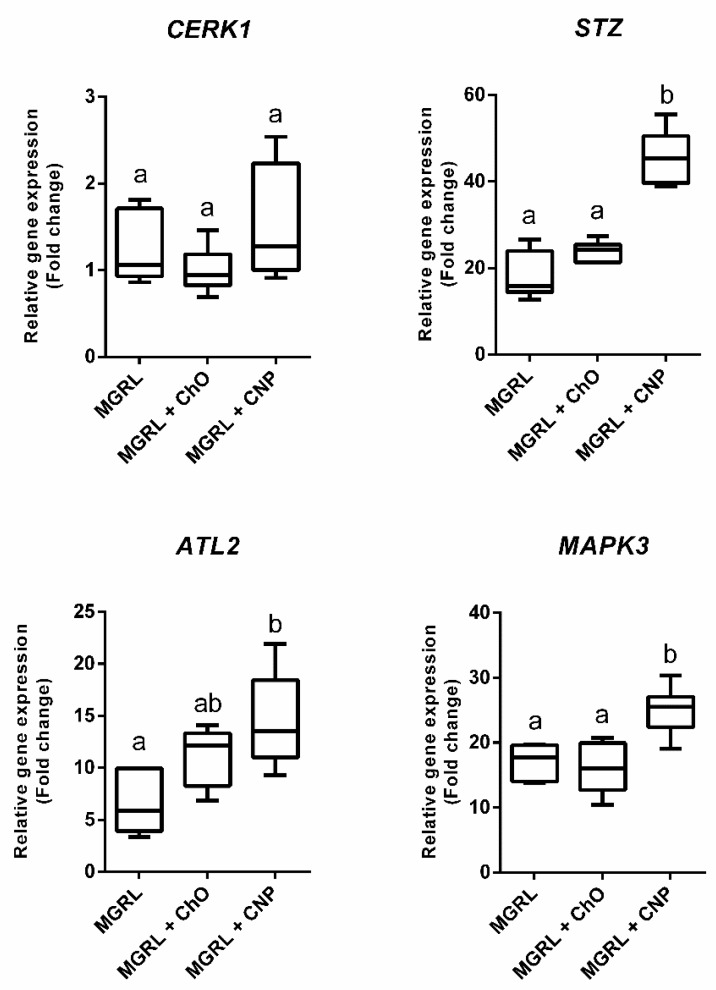
The expression of genes related to early defensive responses induced by chitin in seedlings of *Nicotiana benthamiana* submerged for 1 h in MGRL medium (MGRL), MGRL medium supplemented with 1 mg/mL of chitin oligomers (MGRL + ChO), or MGRL medium supplemented with 1 mg/mL of chitin nanoparticles (MGRL + CNP). Of the analyzed genes, 3 out of 4 increased their expression in all conditions (relativized against the basal expression of seedlings without stimulus, where the fold change value is 1). However, the treatment with CNP generated a greater increase in the expression of *STZ*, *ATL2,* and *MAPK3* genes when was compared to both MGRL and chitin oligomers. The exception was *CERK1* gene, which maintained its basal expression in all treatments. Letters indicate statistical differences between means, according to one-way ANOVA and Duncan’s multiple range test with *p* < 0.01.

The expression of the genes *STZ*, *ATL2,* and *MAPK3* increased when seedlings of *N. benthamiana* were submerged in a liquid MGRL medium (Figure 2). Furthermore, the expression of the genes in seedlings submerged in medium MGRL supplemented with chitin oligomers at 1 mg/mL was similar to the detected with MGRL. However, in presence of MGRL supplemented with CNPs at 1 mg/mL, the increase in the expression of the mentioned genes was significantly greater than the observed with chitin oligomers (Figure 2). This behavior was not observed in the *CERK1* gene, where the expression was similar in treatments with MGRL, CNPs, and chitin oligomers.

### 2.3. ROS Production Increases in Plants Treated with CNPs

**Figure 3:**
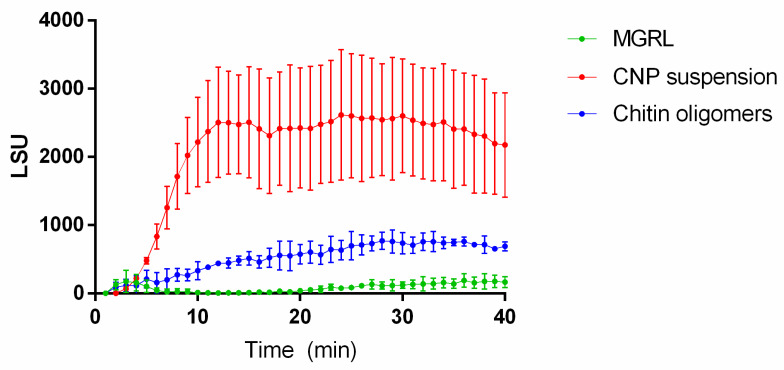
The synthesis of reactive oxygen species (ROS) in seedlings of *N. benthamiana* treated with CNPs and chitin oligomers, measured through chemiluminescence generated by the seedlings during treatments. The chemiluminescence, expressed in luminescence standard units (LSU), increased significantly in seedlings when both forms of chitin are applied. However, the ROS production in CNP suspension was significantly higher than the ROS production mediated by chitin oligomers. Statistical differences were calculated using one-way ANOVA with Duncan’s multiple range test with *p* < 0.01.

ROS production was observed in seedlings of *N. benthamiana* treated with both CNPs and chitin oligomers when compared with the control. However, CNPs were capable of elevating ROS production significantly in contrast to oligomerized chitin (Figure 3), reaching the maximum production at approximately 13 min.

## 3. Discussion

The research on natural and sustainable alternatives capable of replacing the use of fertilizers and synthetic pesticides in crops has driven the development of chitin-based alternatives as an option for new agricultural products. This macromolecule was proven to be both effective in direct control of phytopathogenic microorganisms and as biofertilizers or biostimulants in various production systems [2].

For nanomaterials based in chitin and chitosan, such as nanowhiskers, nanofibers, and chitosan/metal nanoparticles, similar effects were demonstrated to occur on productive parameters compared with non-nanostructured chitin and chitosan; such as effectiveness against pathogens, improvement in the productivity on plants with agricultural importance, and better quality parameters of fruits [20].

In this study, we determined some effects of CNPs generated by emulsion W/O on *N. benthamiana*. The CNPs activated early physiological effects related to defensive responses regulated by chitin, such as the expression of defense genes and the production of ROS. The first step in the generation of CNPs was the dissolution of chitin, which is a highly insoluble polymer [21]. The freeze-thaw methodology described by Hu et al. [22] proved to be adequate for producing a homogeneous chitin solution, which is also suitable for use in the development of W/O emulsions. This methodology allows for the synthesis of particles ranging from approximately 100 to 800 nm in diameter with an average size of 280.3 nm in diameter (Figure 1B). This characteristic is important when the interaction between CNPs and plant tissue is considered because this size could be relevant for contact with external tissue and result in internalization to the plant. For example, in foliar applications, the pore size of the stomata in various plant species varies between 5 and 40 μm long and between 2 and 10 μm wide [23], allowing for the entry of CNPs through the pores and, eventually, their dissemination around tissues adjacent to the pore or through the plant systematically. In roots, nanoparticles are capable of penetrating tissues through the apoplast [24], where the pore size of the cell wall varies between 10 and 20 nm in normal conditions and between 30 to 60 nm when the pectin is damaged [25]. The CNPs are larger than the cell walls pores, which could stop them from penetrating through the apoplast. Although the mechanism of modification, translocation, and mobilization of chitin inside the plant has not been dilucidated yet, the presence of endo- and exo-chitinases in plant tissues [26,27,28] could assist with the processing and modification of CNPs, allowing them to enter the roots successfully.

Our results demonstrated that despite the size of the CNPs, an interaction between the particles and the plant is occurring and that could initiate early defense mechanisms described for non-nanostructured chitin in *N. benthamiana*. This response was measured based on the PTI defensive response model, demonstrating that CNPs can activate early defense mechanisms, either similarly or more efficiently than chitin oligomers, as we exemplified with the increased expression of *STZ*, *ATL2,* and *MAPK3* genes, and the activation of the early first burst of ROS synthesis. The seedlings submerged in suspensions of CNP increased the expression of the genes more efficiently than that submerged in suspension of chitin oligomers (Figure 2), and the CNPs applied in roots triggered a higher production of ROS, in contrast with the chitin oligomers (Figure 3). Although the size of the acid-treated chitin oligomers is not known, the CNPs demonstrated better performance than the molecule without nanostructuration.

For the development of defense signals related to chitin recognition, hydrolysis of polymeric chitin is needed to obtain recognizable oligomeric fragments [20]. As discussed above, we hypothesized that the degradation of CNPs could occur by the action of constitutive chitinases in some tissues of the plant, such as apoplast or guard cells [28]. Additionally, the CNPs induced early responses in the plants faster than chitin oligomers (Figure 2 and Figure 3) due to the probability of better degradation by chitinases or direct interaction with chitin receptors in contrast to non-nanostructured chitin, improving the efficiency of recognition for this elicitor when was nanostructured. This conclusion is consistent with that observed by Egusa et al. [29], where, after the treatment of *Arabidopsis thaliana* with chitin nanofibers, the production of ROS was more efficient in contrast to chitin oligomers between 2 and 6 mer, also demonstrating that the degradation of nanostructured chitin, by chitinases, is more efficient compared to natural chitin.

We demonstrated that the recognition of the CNPs by *N. benthamiana* changed the expression of orthologous genes that were previously described as being activated by chitin recognition in *A. thaliana* [30]. When the plant was conditioned under biotic (CNPs or chitin oligomers) and abiotic (flooding) stress, the analyzed genes increased their expression (Figure 2). Considering that all treatments had flooding as a common stressor, the increment in the expression of genes related to chitin recognition under abiotic stress demonstrated the implication and upregulation of these genes by proteins, signaling molecules, and/or transcription factors related to other types of stress, as occurs with the regulation of genetic expression by proteins related to the WRKY family under biotic and abiotic stress [31]. However, the increase in expression generated by the presence of CNPs after one hour of treatment is significantly higher than that produced by flooding alone and chitin oligomers in most of the genes analyzed, confirming that *N. benthamiana* seedlings can recognize the presence of the CNPs among the abiotic stress quickly and efficiently.

As mentioned above, most genes increased their expression level with CNPs, except for *CERK1*. This gene encodes the CERK1 protein, which is a transmembrane receptor involved in the recognition of chitin oligomers, with a low affinity for its ligand [5]. According to data collected from e-plant (https://bar.utoronto.ca/eplant), based on the vast bibliography from studies in *A. thaliana*, and also described by Ramonell et al. [30], this gene is activated after one hour of incubation with fungal and bacterial elicitors. However, our results demonstrated that changes in its expression and, probably, the synthesis of CERK1 did not occur at 1 h of stress stimulus. This is the first report of this effect on *N. benthamiana* due to the lack of studies about the behavior of gene expression in this plant under chitin-mediated stimuli, and could be associated with a down-regulation of the immune signaling focused in the cellular concentration of this receptor. For example, previous studies demonstrated how in the leaves of *N. benthamiana* the transient production of the *A. thaliana* protein PUB12, that is part of the chitin-activated signaling cascade and interacts with CERK1 and other related proteins, produces a decrease in the cellular concentration of CERK1 and, consequently, a desensitization to chitin [32]. This decrease does not occur by the direct action of PUB12 over the receptor, demonstrating a possible indirect mechanism of *CERK1* regulation that could be related with the expression of the gene, and reveal a possible requirement of the plant cells to regulate the concentration of this receptor during the activation of defensive responses. Future studies should be conducted to corroborate this hypothesis.

In summary, CNP*s* obtained by emulsion are capable of being recognized by seedlings of *N. benthamiana*, activating ROS synthesis and increasing the expression of some genes related to early defensive responses more efficiently than non-nanostructured chitin. Our study demonstrated the potential of using novel nanostructured forms of chitin to improve agriculture, instead of native forms of chitin, due to its difficult application. As an additional effect, nanostructured chitin allows for greater dispersion when used with common solvents. Currently, chitosan, a chemical derivative of chitin, is widely used in agriculture and has demonstrated a direct antimicrobial and bio-stimulant effect on plant systems [33]. Chitosan could be used for an evasion strategy of the plant’s defensive systems by some fungi [34] and even increase the virulence of others [35]. Our experimental results strengthen the support for the use of nanostructured chitin and its elicitor mechanism of action in the area of sustainable agriculture, in addition to the proven activation of chitin-mediated responses in plant models other than *A. thaliana*.

## 4. Materials and Methods

### 4.1. Dissolution of Chitin

A modified method described by Hu et al. [22] was used to obtain an aqueous solution of solubilized chitin. Firstly, a suspension of 2% chitin flakes (Sigma-Aldrich, Saint Louis, MO, USA) in 8% NaOH and 4% urea was prepared. This suspension was subjected to six freeze-thaw cycles (−80 °C to room temperature cycles) to obtain a viscous CS, with some insolubilized fragments that remained in suspension. The CS was centrifuged at 8000× *g* for 15 min and the supernatant was recovered for subsequent CNP synthesis. To determine the concentration of chitin in solution, CS and 1% H_2_SO_4_ (1:1) were mixed into a previously weighed tube to obtain an insoluble chitin gel. The tube was centrifugated at 8000× *g* and the pellet obtained was resuspended in MilliQ quality water and this procedure was repeated twice. Finally, the resulting pellet was lyophilized and weighted in the tube, subtracting the mass of the tube from the value obtained.

### 4.2. Synthesis of Chitin Oligomers

The hydrolysis of the polymer was based on a method described by Revol and Marchessault [36]. A suspension of commercial chitin in 3 M HCl (Sigma-Aldrich, Saint Louis, MO, USA) was incubated at 90 °C for 1.5 h, then centrifuged at 8000× *g* for 10 min, then the supernatant was removed, resuspending the residual pellet in a new solution of 3 M HCl and repeating the procedure twice. At the end of three incubations in HCl, the pellet obtained was centrifuged and resuspended in MilliQ water twice, obtaining a new insoluble pellet and a cloudy supernatant, which contained the chitin oligomers. This supernatant was recovered, dialyzed, and subsequently lyophilized, obtaining a powdered form of the chitin oligomers.

### 4.3. Generation of CNP Using W/O Emulsion

A mixture of CS and hexane was obtained in a ratio of 1:10 and Span 80 was added to a final concentration of 5%. The solution was sonicated at a 35% amplitude for 5 min divided in pulses of 1 min using a Branson 450 Digital Sonifier (Marshall Scientific, Hampton, NH, USA). The solvent was removed using a rotary evaporator at 35 °C until a solid powder was obtained. This powder was resuspended using absolute ethanol and washed three times, and then a pellet was obtained by centrifugation and removal of the supernatant at 9000× *g*. The washed pellet was resuspended in MilliQ water, dialyzed for 24 h in water, and sonicated at 100% amplitude for 10 min in pulses of 2:30 min at a temperature below 25 °C. Chitin content was verified using an aliquot of 1 mL of CNP suspension and centrifuged at 14,000× *g* for 5 min. It was subsequently resuspended in 1 mL of filtered chitinase solution (100 mg/mL of Lysing enzymes from *Trichoderma harzianum* (Sigma-Aldrich, Saint Louis, MO, USA) dissolved in 0.7 M NaCl) and incubated for 3 h at 30 °C with constant stirring at 80 rpm. After incubation, the samples were observed under phase-contrast microscopy. To determine the size and surface charge of the CNPs, a 1:10 dilution of CNPs in MilliQ water was analyzed using a Zetasizer Nano ZS (Malvern PANalytical, Malvern, UK), measuring size, polydispersity, and Z-potential.

### 4.4. Plant Material

Seeds of *Nicotiana benthamiana* were disinfected by soaking them for 10 min in a 0.5% sodium hypochlorite solution under gentle and constant agitation. The disinfected seeds were washed twice with sterile distilled water and incubated in a liquid MGRL medium supplemented with sucrose at 2% [37]. The seeds were incubated at a temperature of 21 °C with a photoperiod of 16/8 h light/dark cycle for 20 days.

### 4.5. Incubation of Seedlings with CNP, Chitin Oligomers, and RNA Extraction

One sample, formed by five 20-day old *N. benthamiana* seedlings, was submerged for 1 h in a new liquid MGRL medium supplemented with CNPs at a concentration of 1 mg/mL. The controls consisted of seedlings submerged for 1 h in a liquid MGRL medium supplemented with: chitin oligomers at 1 mg/mL, a liquid MGRL medium without supplements, and seedlings without stimulus. After incubation, the seedlings were frozen with liquid nitrogen and ground for RNA extraction using TRIzol (Invitrogen, Waltham, MA, USA), following the manufacturer’s instructions. Each experiment was conducted in triplicate.

### 4.6. Primer Design for qRT-PCR

Genes previously described as overexpressed in chitin-treated *Arabidopsis thaliana* plants were used for the search for orthologous genes in *N. benthamiana* (Table 1) [30]. First, protein sequences were obtained from The Arabidopsis Information Resource database (TAIR, https://www.arabidopsis.org/), and subsequently, a search for orthologous genes in *N. benthamiana* was performed using TBLASTN over *N. benthamiana* genome database (https://solgenomics.net/organism/Nicotiana_benthamiana/genome). Table 1 shows the ID of each gene discovered in the *N. benthamiana* genome database, and Table 2 shows the DNA sequences of the primers designed to quantify each gene.

### 4.7. qRT-PCR

A 2 μg of RNA sample from seedlings of *N. benthamiana* was incubated with DNase I (New England Biolabs, Ipswich, MA, USA) following the manufacturer’s instructions. The qRT-PCR protocol consisted of mixing 0.5 μL of DNase-treated RNA per reaction with Brilliant II SYBR Green QRT-PCR Master Mix Kit, 1-Step (Agilent Technologies, Santa Clara, CA, USA), following the manufacturer’s instructions, and using a PCR protocol consisting in: an initial step of 50 °C for 30 min, a second step at 95 °C for 10 min, followed by 40 cycles at 95 °C for 10 s and 60 °C for 10 s. Fluorescence was measured at the last step. The relative expression of each gene was calculated according to the method described by Vandesompele et al. [38], using three reference genes (Table 2), and the seedlings without stimulus as normalizer. To detect a statistical difference, one-way ANOVA followed by Duncan’s multiple range test was realized at *p* < 0.01 using the software Statgraphics Centurion XV (Statgraphics.Net, Madrid, Spain).

### 4.8. ROS Measurement Using Chemiluminescence Assay

The synthesis of ROS was measured using the protocol described by Egusa et al. [29]. Twenty-day-old *N. benthamiana* seedlings were pre-incubated in liquid MGRL medium supplemented with 0.1% sucrose and 100 μM of L-012 (Wako Pure Chemicals, Osaka, Japan) for 2 h at 22 °C in darkness. After the pre-incubation, the seedlings were transferred to liquid MGRL medium supplemented with 0.1% sucrose, 0.1% sucrose and CNP at 1 mg/mL, or 0.1% sucrose and chitin oligomers at 1 mg/mL. The chemiluminescence was determined by counting photons every 1 min for 40 min using a Tecan InfinitePRO M200 (Tecan, Männedorf, Switzerland). Each experiment was conducted in triplicate, and the statistical significance was calculated with one-way ANOVA with Duncan’s multiple range test at *p* < 0.01 using the software Statgraphics Centurion XV (Statgraphics.Net, Madrid, Spain).

## Figures and Tables

**Table 1:** Genes selected for this study and their ID number in The Arabidopsis Information Resource (TAIR) and *Nicotiana benthamiana* genome database.

Name	Function	*Arabidopsis thaliana* ID	*N. benthamiana* ID
*CERK1*	Perception and transduction of chitin signaling	AT3G21630	Niben101Scf01037g04009.1
*MAPK3*	Kinase activated under specific biotic and abiotic stress	AT3G45640	Niben101Scf36191g00003.1
*STZ*	Transcriptional repressor, activated by chitin oligomers	AT1G27730	Niben101Scf06467g01006.1
*ATL2*	RING-H2 transcription factor, activated by chitin	AT3G16720	Niben101Scf06556g10007.1

**Table 2:** Primers designed for the amplification of selected genes in *N. benthamiana*.

Name	Primer	Primer Sequence	Product Length (bp)
*CERK1*	Forward	GGT TAG GAG TTT TCC TTA TCC TAG	111
	Reverse	CCC GCC AAA CAT AGA ATG AAG CTA AAG C	
*MAPK3*	Forward	GTC TGC TCG GTG TTG AAT ACG G	143
	Reverse	CCA ATT ACA TTT TCA TGG TCT AAA TGG CG	
*STZ*	Forward	CCT CGT AGT ATG GAA CGA CAG	145
	Reverse	GCG CGT GTA TTT TCA TCA CTG GAA C	
*ATL2*	Forward	GGG AAG ATA ATG CTA AGC GCT ATT G	145
	Reverse	GGG TAC GGC GAT GGT GGA TCC TAC	
*18S ^a^*	Forward	GCA AGA CCG AAA CTC AAA GG	107
	Reverse	TGT TCA TAT GTC AAG GGC TGG	
*EF1A ^a^*	Forward	AGC TTT ACC TCC CAA GTC ATC	116
	Reverse	AGA ACG CCT GTC AAT CTT GG	
*L23 ^a^*	Forward	AAG GAT GCC GTG AAG AAG ATG T	110
	Reverse	GCA TCG TAG TCA GGA GTC AAC C	

*^a^* Housekeeping genes, described in [39].

## Abbreviations

CNP	chitin nanoparticles
W/O	water in oil
ROS	reactive oxygen species
MAMP	microbe-associated molecular pattern
LPS	lipopolysaccharide
dsRNA	double-stranded RNA
PTI	PAMP-triggered immunity
CS	chitin solution
qRT-PCR	quantitative reverse transcription polymerase chain reaction

## References

[1] Kaya M., Česoniene L., Daubaras R., Leskauskaite D., Zabulione D. (2016). Chitosan coating of red kiwifruit (Actinidia melanandra) for extending of the shelf life. Int. J. Biol. Macromol..

[2] Sharp R. (2013). A Review of the Applications of Chitin and Its Derivatives in Agriculture to Modify Plant-Microbial Interactions and Improve Crop Yields. Agronomy.

[3] Wan J., Zhang X.-C., Stacey G. (2008). Chitin signaling and plant disease resistance. Plant Signal. Behav..

[4] Jones J.D.G., Dangl J.L. (2006). The plant immune system. Nature.

[5] Cao Y., Liang Y., Tanaka K., Nguyen C.T., Jedrzejczak R.P., Joachimiak A., Stacey G. (2014). The kinase LYK5 is a major chitin receptor in Arabidopsis and forms a chitin-induced complex with related kinase CERK1. eLife.

[6] Desaki Y., Miyata K., Suzuki M., Shibuya N., Kaku H. (2018). Plant immunity and symbiosis signaling mediated by LysM receptors. Innate Immun..

[7] Lopez-Moya F., Suarez-Fernandez M., Lopez-Llorca L.V. (2019). Molecular mechanisms of chitosan interactions with fungi and plants. Int. J. Mol. Sci..

[8] Taj G., Agarwal P., Grant M., Kumar A. (2010). MAPK machinery in plants: Recognition and response to different stresses through multiple signal transduction pathways. Plant Signal. Behav..

[9] Wang C., Wang G., Zhang C., Zhu P., Dai H., Yu N., He Z., Xu L., Wang E. (2017). OsCERK1-Mediated Chitin Perception and Immune Signaling Requires Receptor-like Cytoplasmic Kinase 185 to Activate an MAPK Cascade in Rice. Mol. Plant.

[10] Miya A., Albert P., Shinya T., Desaki Y., Ichimura K., Shirasu K., Narusaka Y., Kawakami N., Kaku H., Shibuya N. (2007). CERK1, a LysM receptor kinase, is essential for chitin elicitor signaling in Arabidopsis. Proc. Natl. Acad. Sci. USA.

[11] Shinya T., Yamaguchi K., Desaki Y., Yamada K., Narisawa T., Kobayashi Y., Maeda K., Suzuki M., Tanimoto T., Takeda J. (2014). Selective regulation of the chitin-induced defense response by the Arabidopsis receptor-like cytoplasmic kinase PBL27. Plant J..

[12] Yamada K., Yamaguchi K., Shirakawa T., Nakagami H., Mine A., Ishikawa K., Fujiwara M., Narusaka M., Narusaka Y., Ichimura K. (2016). The Arabidopsis CERK 1-associated kinase PBL 27 connects chitin perception to MAPK activation. EMBO J..

[13] Lohmann G.V., Shimoda Y., Nielsen M.W., Jorgensen F.G., Grossmann C., Sandal N., Sørensen K., Thirup S., Madsen L.H., Tabata S. (2010). Evolution and regulation of the lotus japonicus LysM receptor gene family. Mol. Plant Microbe Interact..

[14] Bozsoki Z., Cheng J., Feng F., Gysel K., Vinther M., Andersen K.R., Oldroyd G., Blaise M., Radutoiu S., Stougaard J. (2017). Receptor-mediated chitin perception in legume roots is functionally separable from Nod factor perception. Proc. Natl. Acad. Sci. USA.

[15] Shimizu T., Nakano T., Takamizawa D., Desaki Y., Ishii-Minami N., Nishizawa Y., Minami E., Okada K., Yamane H., Kaku H. (2010). Two LysM receptor molecules, CEBiP and OsCERK1, cooperatively regulate chitin elicitor signaling in rice. Plant J..

[16] Yamada K., Yamaguchi K., Yoshimura S., Terauchi A., Kawasaki T. (2017). Conservation of chitin-induced MAPK signaling pathways in rice and arabidopsis. Plant Cell Physiol..

[17] Zargar V., Asghari M., Dashti A. (2015). A Review on Chitin and Chitosan Polymers: Structure, Chemistry, Solubility, Derivatives, and Applications. ChemBioEng Rev..

[18] Dietz K.J., Herth S. (2011). Plant nanotoxicology. Trends Plant Sci..

[19] Parisi C., Vigani M., Rodriguez-Cerezo E. (2015). Agricultural nanotechnologies: What are the current possibilities?. Curr. Sci..

[20] Shibuya N., Minami E. (2001). Oligosaccharide signalling for defence responses in plant. Physiol. Mol. Plant Pathol..

[21] Kurita K. (2006). Chitin and chitosan: Functional biopolymers from marine crustaceans. Mar. Biotechnol..

[22] Hu X., Du Y., Tang Y., Wang Q., Feng T., Yang J., Kennedy J.F. (2007). Solubility and property of chitin in NaOH/urea aqueous solution. Carbohydr. Polym..

[23] Eckerson S. (1908). The Number and Size of the Stomata. Bot. Gaz..

[24] Judy J.D., Unrine J.M., Rao W., Wirick S., Bertsch P.M. (2012). Bioavailability of gold nanomaterials to plants: Importance of particle size and surface coating. Environ. Sci. Technol..

[25] McCann M.C., Wells B., Roberts K. (1990). Direct visualization of cross-links in the primary plant cell wall. J. Cell Sci..

[26] Hamid R., Khan M.A., Ahmad M., Ahmad M.M., Abdin M.Z., Musarrat J., Javed S. (2013). Chitinases: An update. J. Pharm. Bioallied Sci..

[27] Collinge D.B., Kragh K.M., Mikkelsen J.D., Nielsen K.K., Rasmussen U., Vad K. (1993). Plant chitinases. Plant J..

[28] Kasprzewska A. (2003). Plant chitinases—Regulation and function. Cell. Mol. Biol. Lett..

[29] Egusa M., Matsui H., Urakami T., Okuda S., Ifuku S., Nakagami H., Kaminaka H. (2015). Chitin Nanofiber Elucidates the Elicitor Activity of Polymeric Chitin in Plants. Front. Plant Sci..

[30] Ramonell K.M., Zhang B., Ewing R.M., Chen Y., Xu D., Stacey G., Somerville S. (2002). Microarray analysis of chitin elicitation in Arabidopsis thaliana. Mol. Plant Pathol..

[31] Chen F., Hu Y., Vannozzi A., Wu K., Cai H., Qin Y., Mullis A., Lin Z., Zhang L. (2017). The WRKY Transcription Factor Family in Model Plants and Crops. CRC Crit. Rev. Plant Sci..

[32] Yamaguchi K., Mezaki H., Fujiwara M., Hara Y., Kawasaki T. (2017). Arabidopsis ubiquitin ligase PUB12 interacts with and negatively regulates Chitin Elicitor Receptor Kinase 1 (CERK1). PLoS ONE.

[33] El Hadrami A., Adam L.R., El Hadrami I., Daayf F. (2010). Chitosan in plant protection. Mar. Drugs.

[34] El Gueddari N.E., Rauchhaus U., Moerschbacher B.M., Deising H.B. (2002). Developmentally regulated conversion of surface-exposed chitin to chitosan in cell walls of plant pathogenic fungi. New Phytol..

[35] Gao F., Zhang B., Zhao J.H., Huang J.F., Jia P.S., Wang S., Zhang J., Zhou J.M., Guo H.S. (2019). Deacetylation of chitin oligomers increases virulence in soil-borne fungal pathogens. Nat. Plants.

[36] Revol J.F., Marchessault R.H. (1993). In vitro chiral nematic ordering of chitin crystallites. Int. J. Biol. Macromol..

[37] Naito S., Hirai M.Y., Chino M., Komeda Y. (1994). Expression of a Soybean (Glycine max [L.] Merr.) Seed Storage Protein Gene in Transgenic Arabidopsis thaliana and Its Response to Nutritional Stress and to Abscisic Acid Mutations. Plant Physiol..

[38] Vandesompele J., De Preter K., Pattyn F., Poppe B., Van Roy N., De Paepe A., Speleman F. (2002). Accurate normalization of real-time quantitative RT-PCR data by geometric averaging of multiple internal control genes. Genome Biol..

[39] Liu D., Shi L., Han C., Yu J., Li D., Zhang Y. (2012). Validation of Reference Genes for Gene Expression Studies in Virus-Infected Nicotiana benthamiana Using Quantitative Real-Time PCR. PLoS ONE.

